# Supplementary material for: Quality of Life and Psychosocial Well-Being among Intersex-Identifying Individuals in Puerto Rico: An Exploratory Study
Source: Int J Environ Res Public Health. 2023 Feb 7;20(4):2899. doi: 10.3390/ijerph20042899 (PMC9957316; doi:10.3390/ijerph20042899)
Supplement: Supplementary file 1 [file ijerph-20-02899-s001.zip › ijerph-2142732-supplementary.pdf]

Supplementary Material:

a.

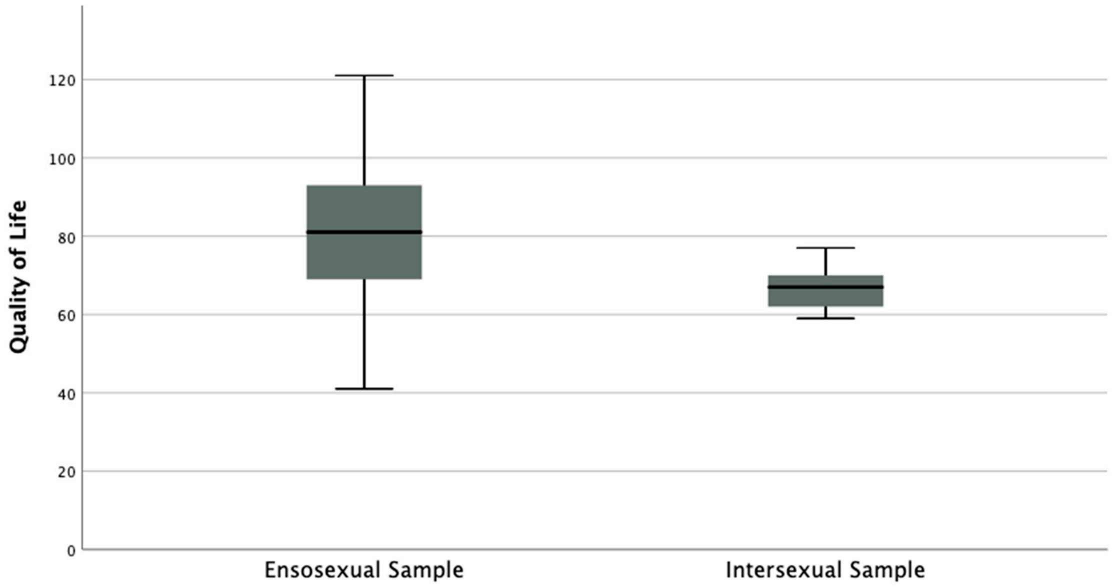

b.

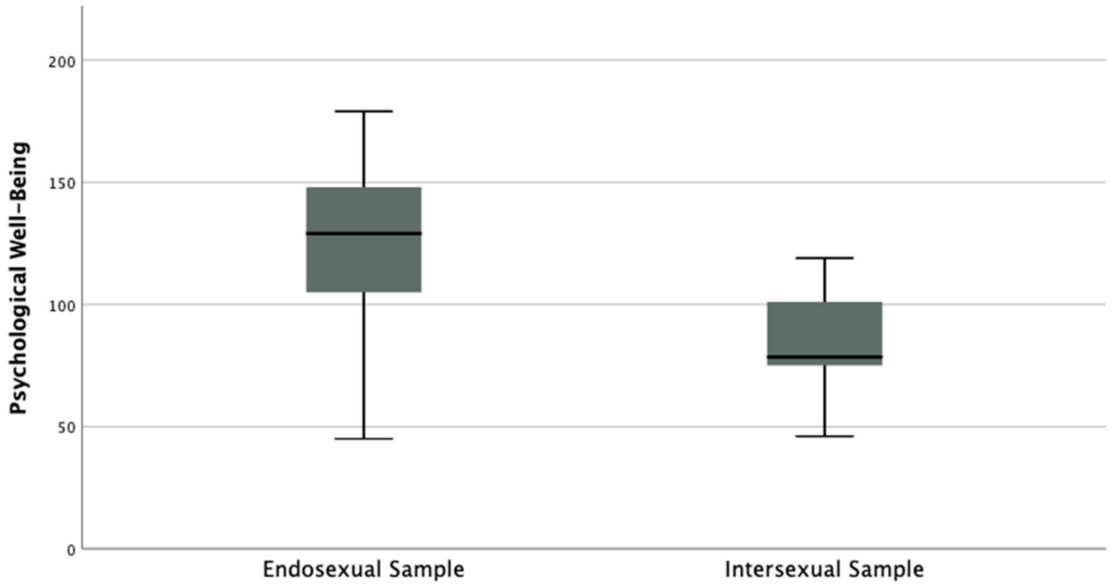

c.

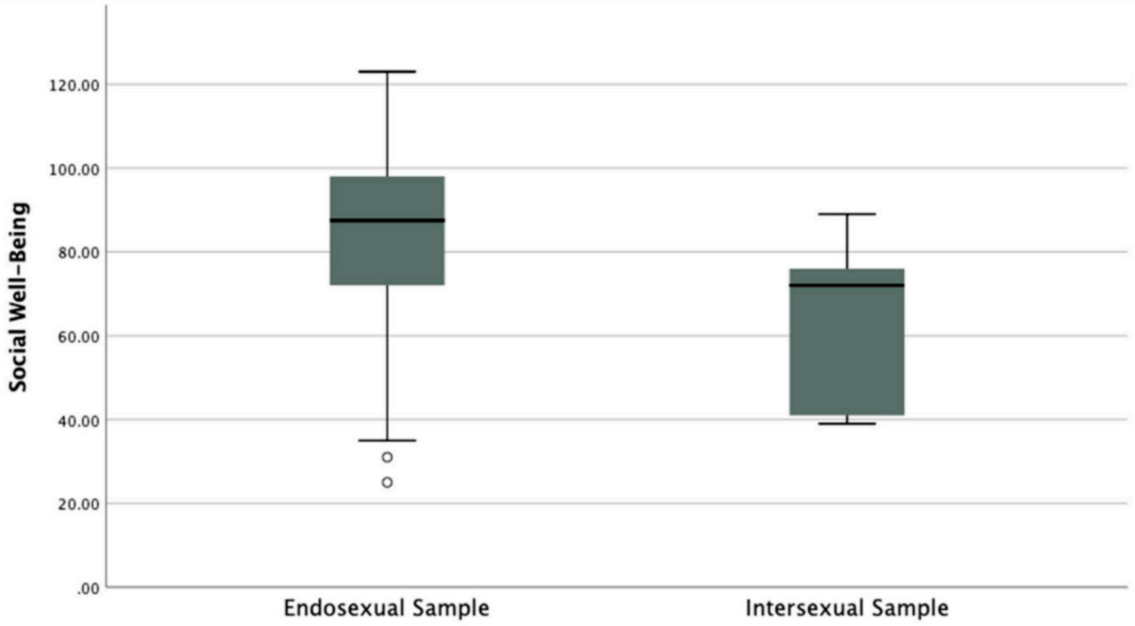

Figure S1: Box Plots and Whiskers for Quality-of-Life (a), Psychological Well-Being (b) and Social Well-Being (c).
